# P-302. Real-world Utilization Management of Antiretrovirals for HIV Treatment and Pre-exposure Prophylaxis: A Systematic Literature Review

**DOI:** 10.1093/ofid/ofaf695.522

**Published:** 2026-01-11

**Authors:** Girish Prajapati, Sean P Fleming, Melissa E Badowski, David E Koren, Orbit R Clanton, Harshil Patel, Lucia Perez-Kempner, Astha Jain, Sugandh Sharma

**Affiliations:** Merck & Co., Inc., Rahway, NJ; Merck & Co., Inc., Rahway, NJ, USA, Philadelphia, Pennsylvania; University of Illinois Chicago, Chicago, Illinois; Temple University Health System, Philadelphia, PA; Columbia University CRS ACTG GCAB CSS, New York, New York; Parexel, London, England, United Kingdom; Parexel International, Alicante, Comunidad Valenciana, Spain; Parexel International, Alicante, Comunidad Valenciana, Spain; Parexel, London, England, United Kingdom

## Abstract

**Background:**

The advent of antiretroviral therapy (ART) has transformed the management of living with HIV into a chronic condition, however, barriers to access persist. Utilization management techniques (UMTs) used by payers to optimize care and resource efficiency often pose challenges for people living with HIV (PLWH) and individuals with increased likelihood of HIV exposure. This study examined the burden of ART-specific UMTs across access pathways in the US, and impact on PLWH, individuals vulnerable to HIV acquisition, and the healthcare system.

**Methods:**

A systematic literature review was conducted in MEDLINE^®^, Embase^®^, JSTOR, PubMed, and government/non-government HIV management websites focusing on ART-specific UMTs in treatment and pre-exposure prophylaxis (PrEP) of HIV in the US. The search covered publications from the last 10 years and conference abstracts from the last 4 years. Studies that met predefined inclusion criteria, based on the PICOTS framework, were included.

**Results:**

Of 1,863 records, 21 met the study criteria, mostly from the private setting (76%). Prior authorization (PA) was the most common UMT identified (71%), followed by step therapy (5%) and quantity limits (5%) for both HIV treatment and PrEP. For oral HIV PrEP, PAs ranged from 2.3% (Northeast) to 37.3% (South). PA-led rejection rates of up to 21% for HIV PrEP regimens were identified, with formulary restrictions contributing to worse clinical outcomes for HIV treatment and PrEP. While there was a paucity of economic burden data for individuals, formulary restrictions imposed substantial costs to the healthcare system ($2.19 billion higher vs. the open formulary). Provider-based studies reported UMTs substantially impact HIV care, with one study reporting that 76% of providers indicate interference with optimal care, 72% citing UMTs and formularies impede prescribing optimal HIV treatment/PrEP.

**Conclusion:**

UMT may pose substantial barriers to timely HIV care and prevention. While intended to control costs, they often result in treatment delays, worsening outcomes, and higher expenses. Despite national efforts to eliminate UMTs for HIV treatment and PrEP, access to ART remains unequal. Policy reforms for UMTs may help facilitate equitable access to HIV care, improve health outcomes, and reduce the HIV burden.

**Disclosures:**

Girish Prajapati, M.B.B.S., MPH , Merck & Co., Inc.: Employee|Merck & Co., Inc.: Stocks/Bonds (Private Company) Sean P. Fleming, PhD, MSW, Merck & Co., Inc., Rahway, NJ, USA: Employee|Merck & Co., Inc., Rahway, NJ, USA: Stocks/Bonds (Public Company) David E. Koren, PharmD, BCPS, AAHIVP, Gilead Sciences: Advisor/Consultant|Merck Sharp and Dohme: Advisor/Consultant|ViiV Healthcare: Advisor/Consultant Orbit R. Clanton, AOS, Global Community HIV Advocate, Merck & Co., Inc: Advisor/Consultant Harshil Patel, BSc, Merck & Co., Inc: Advisor/Consultant Lucia Perez-Kempner, BPharm, MSc, MBS, Merck & Co., Inc: Advisor/Consultant Astha Jain, M.Pharmacy, Merck & Co., Inc: Advisor/Consultant Sugandh Sharma, M.Sc., Merck & Co., Inc: Advisor/Consultant

